# Improving the biological realism of predator–prey size relationships in food web models alters ecosystem dynamics

**DOI:** 10.1098/rsbl.2023.0142

**Published:** 2023-10-25

**Authors:** Kieran J. Murphy, Gretta T. Pecl, Jason D. Everett, Ryan F. Heneghan, Shane A. Richards, Anthony J. Richardson, Jayson M. Semmens, Julia L. Blanchard

**Affiliations:** ^1^ Institute for Marine and Antarctic Studies, University of Tasmania, Hobart, Australia; ^2^ The Australian Centre for Excellence in Antarctic Science, University of Tasmania, Hobart, Australia; ^3^ School of the Environment, The University of Queensland, St Lucia, Australia; ^4^ Centre for Marine Science and Innovation, University of New South Wales, Sydney, Australia; ^5^ School of Mathematical Sciences, Queensland University of Technology, Brisbane, Australia; ^6^ School of Natural Sciences, University of Tasmania, Hobart, Australia; ^7^ CSIRO Environment, St Lucia, Australia; ^8^ School of Science, Technology and Engineering, University of the Sunshine Coast, Petrie, Australia

**Keywords:** cephalopods, functional traits, predator–prey interactions, size spectrum, trophic allometry

## Abstract

Body-size relationships between predators and prey exhibit remarkable diversity. However, the assumption that predators typically consume proportionally smaller prey often underlies size-dependent predation in ecosystem models. In reality, some animals can consume larger prey or exhibit limited changes in prey size as they grow larger themselves. These distinct predator–prey size relationships challenge the conventional assumptions of traditional size-based models. Cephalopods, with their diverse feeding behaviours and life histories, offer an excellent case study to investigate the impact of greater biological realism in predator–prey size relationships on energy flow within a size-structured ecosystem model. By categorizing cephalopods into high and low-activity groups, in line with empirically derived, distinct predator–prey size relationships, we found that incorporating greater biological realism in size-based feeding reduced ecosystem biomass and production, while simultaneously increasing biomass stability and turnover. Our results have broad implications for ecosystem modelling, since distinct predator–prey size relationships extend beyond cephalopods, encompassing a wide array of major taxonomic groups from filter-feeding fishes to baleen whales. Incorporating a diversity of size-based feeding in food web models can enhance their ecological and predictive accuracy when studying ecosystem dynamics.

## 1. Introduction

Predator–prey size relationships, which describe typical prey sizes and how they change as the predator grows, are key drivers of ecosystem energy flow and structure [1]. Size-structured food web models serve as powerful tools to understand ecosystem structure and function [2], and predator–prey size relationships are the building blocks within this modelling framework. However, the prevalent use of simple predator–prey size relationships in marine ecosystem models, assuming all fish have the same functional relationship [3,4], oversimplifies reality. For example, the predator–prey mass ratio (PPMR), representing the ratio of mean predator mass to prey mass, is typically assumed constant and frequently set to 100 for fish predators [3,5]. In reality, predator–prey size relationships are diverse [6–9], with PPMR values spanning eight orders of magnitude when considering taxa across an entire community. Some predators can even consume prey larger than themselves, while others consume relatively tiny prey [8,10,11]. In addition, some organisms maintain relatively consistent prey sizes throughout their lives (e.g. baleen whales eat similar-size krill as they get larger), making a constant PPMR inadequate in representing the diversity of predator–prey size relationships observed. Although representation of differing predator–prey size relationships has improved, particularly for zooplankton [5,12], accurately capturing diverse predator–prey size relationships remains limited, especially for higher trophic level organisms, despite their critical role in predicting both functional relationships and emergent ecosystem structure [13].

Cephalopods serve as an excellent case study to examine the impact of distinct higher trophic level predator–prey size relationships on ecosystem dynamics, as they have considerable influence on ecosystem structure and energy transfer within food webs [14–16], and are commercially important [17]. Most cephalopods' life histories are characterized by high growth rates, which are ecologically linked to feeding [18]. However, their unusual life-history characteristics have led to their underrepresentation in ecosystem models [19].

**Figure 1.**
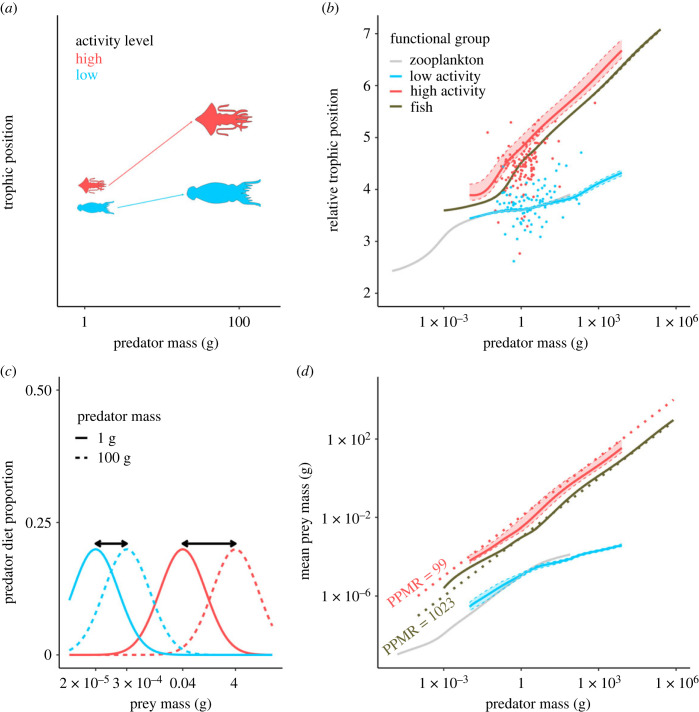
The nature of different predator–prey size relationships and how cephalopods exhibit variations in size-based feeding relative to typical assumptions for ‘average fish’. Schematic representation of cephalopod trophic allometries for low and high-activity functional groups (*a*). Emergent trophic allometries, shown as the median relative trophic position and range for the F + G model, compared with empirical observations from [20] (solid points) (*b*). Distinct predator–prey mass ratios (PPMR) for cephalopod groups displayed by a larger ‘preferred’ mean prey size, as well as larger allometric shift (black arrow) in prey size for high-activity cephalopods (*c*). The ‘realised’ PPMR resulting from distinct feeding traits in our model (*d*). Realised PPMR from the F + G models are compared to empirical values from [6] for a single high-activity cephalopod species (red dotted line, PPMR = 99) and the mean for marine ectotherm vertebrates (dark grey dotted line, PPMR = 1023).

Cephalopod predator–prey size relationships in oceanic ecosystems can be summarized into two groups, defined by body size and activity level. High-activity cephalopods typically exhibit trophic allometry, suggesting a relatively constant PPMR with body size [20]. In contrast, low-activity cephalopods typically maintain a relatively consistent trophic position, suggesting limited change in prey throughout life, which corresponds to an increasing PPMR value as they grow larger [20] (figure 1*a,c*). Although rarely applied to ecosystem models, the latter low-activity predator–prey size relationship may describe many taxonomic groups, including planktivorous fishes, zooplankton, as well some large marine mammals.

Here we explore the consequences of incorporating the two predator–prey size relationship groupings described above on ecosystem structure and function using size spectrum modelling. Specifically, we focus on how adding more realism to feeding diversity and growth impacts abundance–body size structure, ecosystem biomass, production, stability and turnover time. Our findings have important implications when predicting ecosystem responses to perturbations, such as fishing and environmental change [7,21,22].

## 2. Material and methods

### (a) Model description

We extended an existing size spectrum model with functional groups specified using distinct feeding and physiological traits [12]. We modelled a marine community based on empirical sampling during a research voyage on Australia's Marine National Facility *RV Investigator* in the Austral spring of 2017 (electronic supplementary material, figure S1), composed of a phytoplankton size spectrum (as input, electronic supplementary material, a and b, electronic supplementary material, table S1, E9, table S2) and four dynamic functional groups: zooplankton, low and high-activity cephalopods, and fish (electronic supplementary material, table S1). Temporal dynamics of the abundance of each of the four dynamic groups are driven by McKendrick–von Foerster equations with second-order diffusion terms [23]:

2.1 $$\frac{\delta }{\delta t}N_{i}(w,t)=-\frac{\delta }{\delta w}(g_{i}(w,t)N_{i}(w,t))-\mu_{i}(w,t)N_{i}(w,t)+\frac{1}{2}\frac{\partial^{2}}{\partial w^{2}}(\;f_{i}(w,t)N_{i}(w,t)),$$

where $N_{i}(w,t)$ (ind. m^−3^) is the density of individuals in group *i* of weight *w* (g) at time t. Individual growth rates (g yr^−1^) are given by:

2.2 $$g_{i}(w_{i},\;t)=K_{i}V_{i}(w)D_{i}(w,t).$$

$K_{i}$ is the net growth efficiency of group *i*, $V_{i}$ is the search rate of group *i* (electronic supplementary material, E5, table S2), $D_{i}$ is the density of suitable prey (electronic supplementary material, E6, table S2). Individual mortality (yr^−1^) is:

2.3 $$\mu_{i}(w,t)=\mu_{p}(w,t)+\mu_{O_{i}}(w,t),$$

where $\mu_{p}$ is the predation mortality (electronic supplementary material, E7, table S2) and $\mu_{O_{i}}$ is senescence mortality (electronic supplementary material, E8, table S2). Finally, the model includes a diffusion term (g^2^ yr^−1^):

2.4 $$f_{i}(w,t)=y_{i}w^{\alpha i}\sum_{j}K_{ij}^{2}\int_{w_{p}}^{w}{\left(w^{\prime }\right)}^{2}\varphi_{i}(w,w^{\prime })N_{j}(w^{\prime },t)\;dw^{\prime },$$

which accounts for individual demographic variation in growth rates [23].

To assess the impact of cephalopod feeding mode on size-structured communities, we varied their feeding and growth parameters. Empirical values guided the direction and magnitude of parameter variation [18,20,24]. We set high-activity cephalopod PPMR to be lower than the typical 100 used in size spectrum models, by using a value of 25 (see figure 1*c* for a depiction of this parameter). For low-activity cephalopods, we used a modified PPMR equation to account for their lower initial trophic position and slower increase in trophic position observed with increasing predator size [20]. This equation (electronic supplementary material, E2, table S2) allows PPMR to vary across the entire size range of a given functional group, as opposed to the conventional (electronic supplementary material, E1) assumption that PPMR is constant. When PPMR was allowed to vary for the low-activity cephalopods it ranged between approximately 1 × 10^4^ and 3 × 10^6^. By contrast, high-activity cephalopod PPMR was constant at 25 for all sizes. As empirical estimates of cephalopod PPMR are limited, we examined the sensitivity of the model to varied PPMR values for high and low-activity groups (electronic supplementary material, table S1). The range of parameter values considered here are largely based on [20], however we expect our findings to be relevant for other cephalopod communities that exhibit similar trophic and feeding trait patterns, even if the community composition and system types differ [25,26].

To capture higher growth efficiencies typical of cephalopods, we assumed higher net growth conversion efficiencies for the high-activity group (0.3), relative to fish and zooplankton (0.2). These differences are based on empirical values [18,24]. Interspecific values for net growth efficiency between different cephalopod taxa is mainly attributable to differences in active movement [18]. Due to low-activity cephalopods having lower metabolic costs than high-activity cephalopods [24], we assigned low-activity cephalopods a further 10% increase in net growth efficiency (0.4) compared to high-activity cephalopods.

### (b) Simulation experiments

To explore the consequences of cephalopod feeding and growth traits on community dynamics, we designed *in silico* experiments to juxtapose a set of cephalopod-resolved models with a set of control models. The control models included the two cephalopod groups parameterized identically as for fish, except for the minimum, maturation and maximum sizes, while the cephalopod-resolved models included distinct feeding and growth parameters (electronic supplementary material, table S1).

Reproduction is not explicitly resolved in our ecosystem models. Instead, fixed abundances are used at the smallest size of each functional group, which is consistent with established community-level size-structured models [12,27,28]. This assumption of constant recruitment allows a clearer assessment of how the trait distinctions affect community dynamics [12]. To achieve constant recruitment, as in previous models [5,12], the initial abundance of zooplankton at their smallest size class is determined by the abundance of phytoplankton at the corresponding size class (electronic supplementary material, E10, table S2). Similarly, the abundance of fish at their smallest size class is equal to the total abundance of zooplankton in that size class (electronic supplementary material, E11, table S2). The abundance of both cephalopod groups at their smallest size class is set to half the total abundance of zooplankton in that size class.

Model sensitivity in relation to this constant recruitment at the smallest size class was assessed using three abundances for each unique combination of parameters (electronic supplementary material, p_LA*/*HA,_ table S1). This resulted in 27 unique parameter combinations for the feeding and growth (F + G) model set and three for the control model set. Simulations were run for 1000 years with a weekly time step and the dynamic size spectrum discretized into equal 0.1 log_10_ mass (grams) size intervals. After steady state was reached, we use mean values from the final 500 years to describe community metrics and food web output [5].

### (c) Emergent ecosystem properties

We assessed the overall structural effect of different cephalopod predator–prey size relationships on the ecosystem by comparing emergent properties between the different model sets. First, we confirmed that the emergent predator–prey size relationships from the models broadly represent the available empirical values and patterns in the form of relative trophic position (RTP) of the two cephalopod groups [20] and to empirical PPMR values from an aquatic database of individual predator–prey interactions [6]. Then, we examined the effects on abundance–body size structure with slope and intercepts of community size spectra. Finally, to investigate potential effects on energy transfer we compared total community biomass, the coefficient of variation (CV) of biomass, community production, and turnover time. The CV was calculated using the total annual biomass, summed across the four functional groups, for the final 500 years of the model runs. Turnover time was calculated as the inverse of the production to biomass ratio (electronic supplementary material, E13–E15, table S2).

## 3. Results

Including distinct predator–prey size relationships (figure 1*a,c*) produced markedly divergent energy pathways for both cephalopod groups. High-activity cephalopods fed at higher trophic positions and on larger prey sizes compared to other groups (figure 1*b,d*). By contrast, low-activity cephalopods followed an alternative trophic allometry, with a lower trophic position and relatively low rate of change in trophic position with increasing body mass. The relative effects of the distinct predator–prey size relationships appear to be more influential on changes to ecosystem dynamics when comparing feeding traits only to feeding + growth traits combined (electronic supplementary material, figure S4, figure S5). These distinct trophic roles that emerged in the F + G model set were more consistent with empirical observations for the cephalopod functional groups from [20] compared to the control models (root mean square error (RMSE) = 0.87 versus RMSE = 1.31) (figure 1*b*).

**Figure 2.**
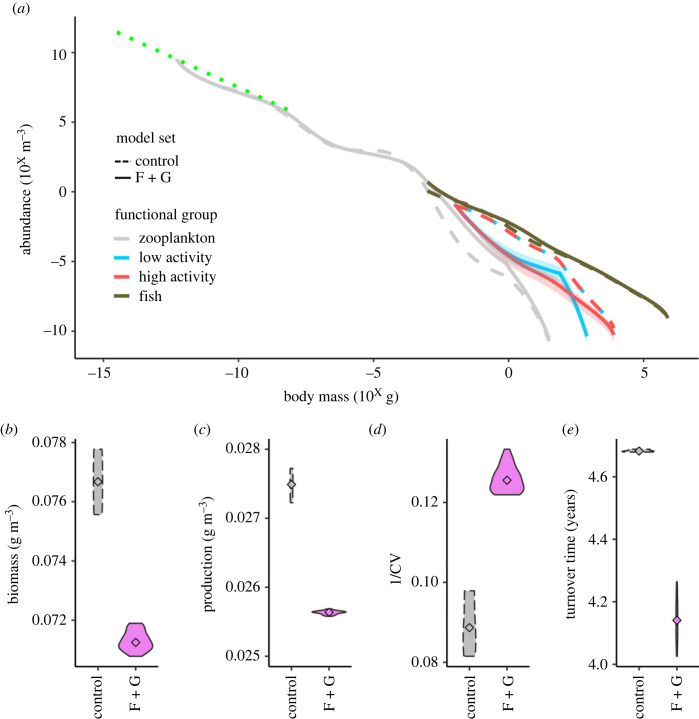
Consequences for ecosystem structure and function of including distinct predator–prey size relationships, via cephalopod feeding and growth traits. Median values and range for the F + G model set abundance size spectra compared to the control models (*a*), and violin plots of the values for each ecosystem across the control (dashed line, grey fill) and F + G (solid line, violet fill) model sets for community biomass (*b*), production (*c*), the inverse coefficient of variation of biomass (1/CV) (*d*), and biomass turnover time (*e*). Variation in panels (*b*–*e*) is a result of testing a range of abundances at the smallest size class for the cephalopod groups for the control model set, and a combination of the initial abundances combined with the range of PPMR, feeding mode, and growth values in the F + G model set.

The divergent trophic patterns of the cephalopod groups affected ecosystem structure and function. The F + G models produced markedly different abundance size spectra for the zooplankton and cephalopod groups, but similar overall community size spectra (figure 2*a*, electronic supplementary material, figure S2). The most pronounced effect of resolving cephalopod traits in the models was the impact on cephalopods themselves, especially the high-activity group, which due to their faster growth rates (electronic supplementary material, figure S3*b*), resulted in a lower standing stock of abundance and biomass.

Overall, resolving these divergent energy pathways for cephalopods led to a lower overall ecosystem biomass and production, both declining 7%, but increased biomass stability by 41% and increased the rate energy turnover by 12% (figure 2*b–e*). Despite the increased stability of community biomass, the stability of the functional groups responded differently to the distinct feeding traits, with cephalopod functional groups exhibiting reduced stability in the F + G models (electronic supplementary material, figure S4). While community production decreased in the F + G models compared to the control, largely explained by lower zooplankton production and to a lesser degree lower cephalopod production in the F + G models, fish production increased (electronic supplementary material, figure S4).

## 4. Discussion

Improving biological realism in a size-structured ecosystem model, through empirically derived distinctions in cephalopod feeding and growth traits, reduced community biomass, increased stability and accelerated biomass turnover, when compared to our control model set. Many other taxonomic and functional groups display similar distinctions in feeding traits as cephalopods, which are not well represented currently in size-structured models. Therefore, our results suggest that this lack of realism in predator–prey size relationship representation in ecosystem models may lead to inaccurate understanding of ecosystem structure and function.

Our results also support the idea that cephalopods can substantially influence marine ecosystem dynamics [29]. The importance of cephalopods providing alternative energy pathways in marine ecosystems has long been recognized and has recently received greater scientific attention due to an apparent rise of cephalopod fisheries and abundance in many ecosystems [16,17,30]. Due to ‘slow energy’ channels being a stabilizing feature of many food webs [31], we were surprised that the inclusion of ‘live fast, die young’ life-history groups had stabilizing properties. This is due to the overriding affect that the diverse size-based feeding traits had relative to growth efficiency traits (electronic supplementary material, figure S4–S5). Whether or not these findings would extend to all cephalopods and to other taxa remains an open question.

Previous work has shown that diverse zooplankton feeding traits affect biomass and transfer efficiency in global ecosystem models [5,12]. While size spectrum models that incorporate diverse life-history traits have been shown to confer stability, this has not yet been studied in the context of alternative predator–prey size relationships that allow PPMR to vary with predator size (including organisms that target the same prey size classes throughout their life). Our findings suggest that improving our understanding of variation in size-dependent feeding within marine ecosystems is likely important for accurate prediction of ecosystem responses to environmental change. A greater focus on incorporating biological realism into the representation of higher trophic-level organisms would likely yield positive outcomes. Investigating the metabolic and movement traits of predators holds great potential for comprehending how predator–prey size relationships scale at the ecosystem level [32]. Given the connection between these traits and activity level groupings in our study, this trait-based approach could offer a viable strategy to enhance the biological realism of size-structured food webs, especially when dealing with limited data availability. In addition, we have shown that size spectrum modelling provides a useful framework for assessing how ecosystem-scale metrics can be driven by the diversity of size-based feeding that is prevalent across individuals within marine communities.

## Data Availability

The data and code associated with this study are available from the Zenodo Repository: https://doi.org/10.5281/zenodo.8241472 [33]. Supplementary material is available online [34].
